# Collectanea Medica: Consisting of Anecdotes, Facts, Extracts, Illustrations, &c.

**Published:** 1820-12

**Authors:** 


### COLLECTANEA MEDICA:

CONSISTING OF

### ANECDOTES, FACTS, EXTRACTS, ILLUSTRATIONS, &c.

*Relating to the History or the Art of Medicine, and the Auxiliary Sciences.*

Floriferis ut apes in saltibus omnia libant,

Omnia nos itidem depascimur aurea dicta.

---

*Extracts of the Report from the Select Committee of the House of Commons on the Doctrine of the Contagion of the Plague, in* 1819.

*Martis*, 29° *die Aprilis*, 1819.

Sir John Jackson, Baronet, in the Chair.

Dr. Robert Tainsh, called in; and Examined.

YOU were surgeon of the Theseus, I believe, in the Mediterranean, at the siege of Acre?—Yes, I was.

In what year was that?—It was in the years 1798 and 1799.

Did you see any cases of plague whilst there?—Yes.

How many cases of plague did you see?—I saw five cases of plague.

Were they on-board the Theseus?—Yes.

What countrymen were the persons infected?—There were three Englishmen and two Frenchmen.

Did they belong to your ship?—The three Englishmen had belonged to the Theseus formerly, and they had been taken in one of our gun-boats. They all appeared infected with the disease.

In what state of the disease were they; had they buboes?—Only one of them had it of any great consequence. The other four were petechial slight cases. They were in a state of convalescence, and had rather the remains of the disease than any thing else. One of the Frenchmen, however, had buboes, and in a state of suppuration.

Did that Frenchman recover?—Yes.

After what lapse of time?—In about three weeks or a month. Both the Frenchmen were sent on shore.

But was there any communication of the plague to any other persons on-board the Theseus?—No.

Were they any means of separating these plague patients from the rest of the crew?—Certainly; all of them were removed into one birth on the starboard side of the ship, called the *sick bay*. Every part of their clothes that were on them on their coming on-board, were immediately taken off and thrown overboard, by my orders. Their bodies were shaved, and no hair left on them that could lodge moisture. They were then washed with soap and warm water, and new clothes were given to them: they had new bedding, new shirts, and every thing new within ten minutes after they came on-board.

Who washed them?—One man whom I persuaded so to do, by handling the patient myself, and applying the poultices to the buboes.

Had you communication with the ship's crew at the same time?—Yes; I examined them every morning, to see whether they had caught any infection, but none of them had.

Did the man who attended these patients mix with the crew?—He communicated generally with the crew of the ship.

What was your object in destroying their clothing in which they came on-board?—It was a mere caution to prevent those clothes from being again used. It was on account of no particular theory, but merely for cleanliness.

It did not arise then from the belief that it was of itself contagious?—No; and, my hands being so much engaged with the knife at that time, I had not much opportunity of studying the disorder.

Did you touch the man who was so badly affected?—Yes, I applied his poultices.

Did the same man attend upon him who had shaved him?—No, the barber of the ship shaved him; and the only precaution used was, when the attendants came out of the sick bay, I made them immerge their hands in a bucket of vinegar.

Do you suppose they always did that?—They were ordered to do so, and a sentry was placed there to oblige them so to do.

How near was the sentry to them?—He was on the opposite side of the ship, the sick bay generally taking in half the gally.

